# Disabled Body‐Minds in Hostile Environments: Disrupting an Ableist Cartesian Sociotechnical Imagination with Enactive Embodied Cognition and Critical Disability Studies

**DOI:** 10.1007/s11245-024-10080-5

**Published:** 2024-08-22

**Authors:** Janna van Grunsven

**Affiliations:** Delft University of Technology, Jaffalaan 5, 2628BX Delft, The Netherlands

**Keywords:** Ableism, Technoableism, Disability, Enactivism, Affordances, Cartesianism, Hostile Environments

## Abstract

A growing body of literature in the field of embodied situated cognition is drawing attention to the hostile ways in which our environments can be constructed, with detrimental effects on people’s ability to flourish as environmentally situated beings. This paper contributes to this body of research, focusing on a specific area of concern. Specifically, I argue that a very particular problematic quasi-Cartesian picture of the human body, the human mind, what it means for these to function well, and the role of technology in promoting such functioning, animate our Western sociotechnical imagination. This picture, I show, shapes the sociotechnical niches we inhabit in an *ableist* manner, perniciously legislating which body-minds have access to a rich world of affordances and are seen as agential and valuable. Because the ableist quasi-Cartesian commitments animating our Western sociotechnical imagination are problematic and pervasive, I argue that exposing and reimagining these commitments should be a prime focal point of those working at the intersection of science, technology, and human values. I present insights from enactive 4E cognition and critical disability studies as fruitful resources for such much-needed reimagining. I also make the case, more provocatively but also more tentatively, that the ableist view of bodily and minded well- functioning animating our Cartesian Western sociotechnical imagination is not only damaging to embodied minds who deviate from the presumed norm, creating inaccessible worlds for some of us; it is in fact a threat to human and planetary flourishing at large.

## Introduction

The sense-making lives of human beings are pervasively shaped by technology. Many of the tools and infrastructures that we engineer support or “scaffold” the kinds of cognitive, affective, and social lives we are able to lead (Sterelny 2010). Crucially, though, what counts as a supportively scaffolded environment (or “niche”) for some may count as a pernicious, maladaptive one for others. This “dark side” of niche-construction (Coninx 2023) is increasingly recognized in the field of embodied situated cognition. A growing body of literature has been drawing attention to the hostile ways in which our sociotechnical niches can be constructed and the detrimental effects this can have on people’s ability to flourish as environmentally situated beings (Slaby 2016; Van Grunsven and IJsselsteijn 2022; Dokumaci 2023; De Carvalho and Krueger 2023; Timms and Spurrett 2023; Osler 2024). This paper contributes to this body of research, focusing on a specific area of concern, namely how *ableist* habits of mind are animating our Western sociotechnical imagination, contributing to sociotechnical environments that are emphatically hostile to disabled people and, arguably, to planetary flourishing at large.

By *sociotechnical imagination* I mean the ways in which we (as individuals and as a society) imagine that developments in science and technology contribute to human, animal, and planetary flourishing (Jasanoff and Kim 2015). What I argue, is that a very particular problematic picture of the human body and mind animates our (Western) sociotechnical imagination. What is more, this picture, and its problematic implications, are quite literally materialized into the world we inhabit, informing what technological artefacts and systems get developed and legislating whose bodies and minds belong. Because the picture of the human mind and body animating our Western sociotechnical imagination is both problematic and pervasive, exposing and reimagining this picture should be a prime focal point in research located at the intersection of science, technology, and human values. I present insights from enactive 4E cognitive science and critical disability studies as capable of providing fruitful resources for such much-needed reimagining.

I argue that the picture of human embodiment animating our Western sociotechnical imagination is problematic in three regards. Firstly, it reflects an ontologically flawed *quasi-Cartesian* view of the human body and, relatedly, the mind. I invoke the term ‘quasi’ here to indicate that I am not interested in thorny ontological debates about how, say, Descartes’ view of the body and the mind in the sixth meditation holds up against my characterization here.^1^ Rather, I am interested in a number of tenets that can be fairly associated with Descartes’ heritage (for the sake of brevity I will, from hereon, drop the ‘quasi’ and simply speak of Cartesianism). Now, one might think that reflecting on Cartesian tenets is hardly worth our time—their bite and significance emptied out after centuries of familiar philosophical arguments have carved out well-trodden anti-Cartesian paths. However, as I aim to show, Cartesianism is alive and kicking in our Western Sociotechnical Imagination, informing what and how we innovate for human bodies and minds and how the desirability of technological innovations are framed by tech companies and the media. As such, identifying these tenets and tracing them back to Descartes is helpful not only because it captures something of the historical depth of this Western way of thinking, but also because it serves as a step towards moving into a different direction. That is, attending to Cartesianism helps to set up a clear contrast with the alternative ontological picture of the human body and mind (or, rather, the embodied-mind), that I will turn to in an effort to redirect our quasi-Cartesian sociotechnical imagination. This alternative picture is grounded in enactive embodied cognition, which is emphatically anti-Cartesian.

The Cartesian picture of the body (and, relatedly, the mind) that I am concerned with, although ontological in the first instance, is not without ethically pernicious implications. This gets me to my second objection. I will argue that Cartesianism informs and props up a pernicious *technoableist* sociotechnical imaginary. Ableism, briefly put, is the valuing of some bodies and minds, the ‘able-bodied’ one’s, over others—where an “able-bodied … person” can be understood as “a body with a set of given functions, skills and properties, which are steered by a central command unit—the consciousness—which is situated in the head” (Moser’s 2000, 5). *Techno*ableism, which is a term coined by STS scholar and disability rights activist Ashley Shew, refers to “a rhetoric of disability that at once talks about empowering disabled people through technologies while at the same time reinforcing ableist tropes about what body-minds are good to have and who counts as worthy.” (2020, 43).

Technoableism is particularly rampant surrounding hyped emerging technologies (such as exoskeletons, social robots, and CRISPR_Cas9 gene-editing), which are often showcased in popular media for their “remarkable” abilities to “restore lost abilities” and “profoundly improve” the “inactive” “disordered” “dependent” lives of disabled people. In fact, many of these technologies materialize into our sociotechnical niches a narrow exclusive view of which body-minds are good to have, shaping the daily use-objects, systems, and infrastructures with(in) which we dwell and the habitual patterns of action and perception enacted within our environment (Hamraie and Fritsch 2019; Van Grunsven and IJsselsteijn 2022; Dokumaci 2023). Shew warns that unless we explicate and critique such technoableism, engineers will very likely continue to develop technologies that undermine the very societal good that they aim to contribute to: where they take themselves to be ‘helping’ disabled (and also ageing) embodied-minds, they are in fact legislating a material-technological space that marginalizes and excludes the very people they take themselves to be designing for.

This, by itself, warrants my plea for a sustained attention to how ideas about human embodiment get tangled up with our sociotechnical imagination. Those of us who work at the intersection of science, technology, and human values simply need to do *much* better when it comes to exposing the rampant ableism in technology development, in order to honor and do justice to the diversity of ways in which human bodily lives can and deserve to be lived. But, and here is my third point, I want to try and expose an additional layer of ethical concern. I will make the case, though somewhat tentatively, that the ableist view of bodily and minded well-functioning entangled with our Cartesian Western sociotechnical imagination, is not only damaging to embodied minds who deviate from the norm, creating inaccessible worlds for some of us; it is in fact a threat to human and planetary flourishing at large.

My argument takes the following steps: I first introduce the tenets of Cartesianism that I am concerned with (Sect. 2). I then show how these tenets inform our Western sociotechnical imagination in a manner that props up a technoableist outlook (Sect. 3). One of the problems with technoableism is that it systematically sidelines the lived experiences of disabled people, a tendency that, I argue, is reflective of a Cartesian posture towards the body, the mind and what counts as scientifically relevant knowledge. In short, Cartesianism prioritizes that which can be known from a detached scientific point of view and cannot take seriously the (disabled) body as the site of rich experiential sense-making and embodied know-how. To get away from this Cartesian tendency, we need a different account of the body. This is where I turn to enactive and affordance-theory insights from the field of embodied situated cognition. I show that these insights not only open up a much-needed reimagining of human embodiment, they also help to explain how technoableism deeply permeates our everyday ways of perceiving disabled embodiment and the world at large, contributing to a phenomenon that I call *the vicious cycle of Cartesian technoableism*. After I discuss how enactivism, infused with critical disability studies, can help break this vicious cycle, I conclude the paper by arguing, tentatively, that this is needed not only to promote more accessible worlds for disabled people but, more generally, to promote human and planetary flourishing at large. To make this case, I build upon recent works from Arseli Dokumaci (2023) and Shew (2023).

## Identifying Quasi-Cartesian Tenets

Let’s begin by looking at some of the specifics of the Cartesian outlook that I am targeting. Familiarly (and very briefly put), for Descartes, the body is understood as a machine-like object, a complex arrangement of matter devoid of any kind of interiority. In the words of Dalia Judovitz: “decontextualized from its worldly fabric, the Cartesian body … cease[s] to function by reference to the human, since its lived, experiential reality … [is] supplanted through mechanical analogues” (1998, 21). The mind, which is theorized as ontologically distinct from the body, is seen as the locus of the self -understood as a radically independent self-sufficient rational thinking thing. Employing a methodological individualism, Cartesianism maintains that the human mind and the human body–*what they are and how they function*–can be understood in their ontological essence without reference to each other or the wider material, physical, and social world. There is a normativity at work in this picture: the Cartesian body and the Cartesian mind function optimally, *as they should*, when they work independently from one another, with the body functioning as a well-oiled complex machine, and the mind abiding by clear rational principles that remain unencumbered by bodily processes and experiences.

I won’t discuss Descartes’ nearly unintelligible account of how his mechanistic machine-like human body and the immaterial thinking mind connect (he offers a location, the pineal gland, not an explanation). What is worth highlighting, though, is that insofar as the body is connected to the mind and to the extent that the body intermingles with the mind’s efforts of understanding the world, it is primarily seen as a source of error, muddling whatever clear, distinct, superior knowledge the mind is allegedly capable of achieving if it were to confine itself to that which can be known from a rational disembodied, and thus allegedly objective, point of view.

Exhibiting an unbridled technological enthusiasm, Descartes imagined that the objective rational scientific knowledge about the physical world that this de-worlded disembodied subject is allegedly capable of acquiring, would provide endless benefits–enabling a *mastery* over bodily life and the natural world that he deemed necessary for human flourishing. In his words,

“[T]o … render ourselves … masters and possessors of nature … is desirable not only for the invention of an infinity of devices that would enable one to enjoy trouble-free the fruits of the earth … but also principally for the maintenance of health, which unquestionably is the first good and the foundation of all the other goods of this life. … one could rid oneself of an infinity of maladies … and even perhaps also the frailty of old age, if one had a sufficient knowledge of their causes and of all the remedies that nature has provided us.” (Descartes 1998, Discourse on Method, Part 6).

Of course, very few (if any) philosophers working on mind and cognition today accept Descartes’ ontology.^2^ At the same time, it is hard to overestimate its formative effect on Western thought. Drew Leder argues for instance, that “Cartesianism,” though developed nearly 400 years ago, has had “profound effects” on the field of medicine, where, in his words:

“The sense of the patient as a living, experiencing, suffering person has been systematically truncated by the model of the body-as-machine, a model that has shaped our understanding of disease, our modes of professional training, diagnosis, and treatment, even the offices and hospitals in which medicine is practiced” (Leder 1992, 33; See also N.D. Jewson 2009).

That is to say, theoretical ontological constructs, even when outdated and largely discredited, can linger and continue to have far-reaching real-life consequences. As I will argue in the next section, this Cartesian influence extends beyond the field of medicine, informing our thinking more broadly about technological innovation and intervention, especially, though not solely, when it concerns the alleged promotion of human minded and bodily flourishing.

## Cartesianism and Ableism

In the introduction I cited Ingunn Moser, who defines “an able-bodied … person [a]s … a body with a set of given function, skills and properties, which are steered by a central command unit –the consciousness—which is situated in the head.” She adds that “Agency, mobility, the ability to communicate verbally, to make discretionary judgments, make decisions and implement them—is thus located in the body … [or] in the self [somehow] residing in that body.” (2000, 5). The link between Ableism and Cartesianism is nearly undeniable in Moser’s characterization. Indeed, the influence of Cartesianism on our sociotechnical imagination becomes repeatedly clear when one starts to pay attention to how disability is typically understood in contexts of technological innovation. We are unphased, perhaps even delighted, by popular science and technology articles suggesting that:

Exoskeletons could “transform the lives of disabled people” in ways that “could help disabled people to be more active” with “Technology … reaching the point where those who have been disabled can be re-enabled” (*The Guardian*).^3^

or that “CRISPR can edit out autism traits,” which “could one day revolutionize the therapies that treat autism and improve the lives of thousands of people who suffer from the developmental disorder” (*Newsweek*).^4^

or that “wearable and go-with devices” ought to be embraced as “tech that could confront the crisis in aging,” enabling aging adults to live with “more independence,” improving “health, safety and independence.” (*VentureBeat*).^5^

When you delve into the articles behind these headlines (and there are many like these), you’ll run into several Cartesian tenets in how disabled (and aging) bodies and minds are framed. Recurring themes and assumptions are that:

1. To be disabled just is to have a mind or body that isn’t functioning as it ought to, where bodies ought to function essentially as well-oiled smooth-running machines such that we can live maximally independent lives, ‘free of an infinity of [bodily] maladies’ and ‘the frailty of old age.’
2. Disability is to be understood and treated at the level of individual bodies and minds. This reflects a Cartesian methodological individualism. Autistic ‘traits’ located inside the autistic person are meant to be ‘therapeutically’ removed from individual bodies; the ‘crisis’ of ageing is to be dealt with by restoring mobile ‘independency’ to frail bodies; allegedly ‘inactive’ disabled bodies must be ‘re-enabled’ with exoskeletons. In the field of critical disability studies, this posture (which locates disability squarely on the part of individual minds and bodies and sees disability as needing to be fixed or cured) is referred to as the medical model of disability. I am here linking this posture to a Cartesian frame of mind.
3. Independence is contrasted with dependence and hailed as the marker of a (productive) life worth living.^6^
4. Technology is here to save the day, raising the lives of disabled persons out of the deplorable inactive state of dependency they are allegedly in (thus echoing Descartes’ technological enthusiasm).
5. In this oh so noble pursuit, it is first and foremost the knowledge of those well-versed in the practical application of science, *not* the lived embodied know-how of disabled people, that guide efforts to ‘support’ disabled lives. This aligns with a Cartesianism skepticism towards the body, which is seen as primarily a source of epistemic error rather than an epistemically valuable site of knowledge acquisition.

To be sure, all sorts of technologies play a crucial role in the lives of all sorts people (disabled and nondisabled alike) and it is far from my intention to suggest that we should no longer invest in technologies that can assist, heal, and comfort us as we live our precarious bodily lives. Nor is it my intention to deny that there is real value in having a sense of independence or that we can experience our own body as getting in the way of that in deeply debilitating ways. Having grappled myself with a form of social anxiety that is entangled with a condition called idiopathic craniofacial erythema (which is just a fancy medical term for excessive and often unprovoked facial blushing likely caused, in part, by an overactive sympathetic nervous system), I am not unfamiliar with the debilitating experience of a non-compliant body and the desire for a medical-technological fix. Nor do I mean to deny that some, perhaps even many of such fixes–when safe, responsive to the needs of actual users, and (financially) accessible–can be conducive to one’s flourishing as a precarious bodily being.

What I want to signal, though, is that we should be deeply mindful of ableist habits, beliefs, and expectations informing how we develop, assess, market, and financially support technological innovations allegedly conducive to human embodied flourishing. We overvalue a body-mind under control and too readily see it as something that ought to be fixed with technology when it isn’t. As Ashley Shew warns, this outlook is both ontologically unrealistic and ethically harmful:

“The technologized disabled body - the re-enabled body, “triumphant” over its own conditions—is a lie. Technology cannot transcend the meatsack; the body is still there, still felt, still handled, enduring. But technology—and the normative ideas of what it means to have the correct body (or mind)—increasingly separates our selves from the bodies with which we encounter the world. … disabled people often need to (re-)integrate our selves with our bodies, while living in a world that instead tries to force us into a fake normality” (2023, 74).

I think most of us are prone to be taken in by this push for fake normality. Think of the enthusiasm you may feel in response to images and headlines lauding the “triumph”^7^ of a paraplegic young man operating an (unaffordable) exoskeleton to kick off the world cup, reassuring us that ‘our’ world, organized around uprightness and bodies that can walk, is desirable and available to all of us thanks to technology. Those among us who work in contexts of engineering and innovation (whether in industry, research, or education) may use such imagery to appeal to the sociotechnical imagination of the public, of committees on funding schemes, or of our students. We thereby further sustain and legitimize a technoableist outlook on human embodiment and flourishing. The late bioethicist and wheelchair user Bill Peace exposes the shallowness of such imagery with effective snark:

Your typical bipedal person exposed to a barrage of misleading news stories is led to believe all paralyzed people share one goal in life--walking. Please cue the soaring inspirational music accompanied by the brave and noble young man or woman struggling to walk surrounded by health care professionals, computer scientists, and engineers who share the same *ritualized ideal* … Come on bipeds, get over yourself. Think and imagine what life can be. Stop obsessing over walking and use your creative mind (2015)

In fact, the ritualized ideal that Peace takes issue with hides a much more complicated story about how factors such as heat-induced swollen sweaty limbs, or uneven un-cared for sidewalks, or snowy and icy weather conditions, or fluctuating body weight, or financial cost and uncooperative insurance companies, affect the experience and desirability of such innovations (See Shew 2023). Furthermore, this ritualized ideal jumps over the not insignificant fact that many people who actually use a wheelchair to get around find the artefact *itself* perfectly suited to that end. In fact, artist and disability rights activist Sue Austin creatively invites us to see that wheelchairs are in fact better understood as *power* chairs that are perfectly suitable to a whole host of desirable ends, such as creating art and deep-sea diving among the ocean’s coral reefs.^8^ It isn’t so much the inability to walk that is necessarily bad *in itself*, but the fact that our sociotechnical niches are often inhospitable to wheelchair users, or the fact that some insurance companies don’t cover treatments for sepsis-causing pressure wounds (which is how Bill Peace died).

There is a deeply troubling mismatch between the actual lived experiences of disabled people and the ways in which disabled bodies are depicted, framed, imagined in contexts of scientific research, technological innovation, and the news stories lauding certain scientific and technological advances (this is a recurring key theme in the works of disability rights activists such as Ashley Shew, Mel Baggs, Harriet McBryde Thompson, Alice Wong, Eli Clare, Donna R. Walton, and so on).^9^ This mismatch is reflective of one of the Cartesian tendencies I discussed earlier: by framing the body as a machine devoid of interiority, Cartesianism cannot take seriously the body as the site of rich experiential sense-making and embodied know-how. To get away from this Cartesian tendency, we need a different account of the body. This is where I now turn to so-called *enactive* insights from the field of 4E Cognition paired with insights from affordance-theory.

## Enactive 4E Cognition: Reimagining the Body as a Site of Sense-Making

In Sect. 2 we saw that Descartes defines the human body as a complex machine devoid of interiority. On his view, there is no ontological difference between the human body or any other kind of physical material substance (there is only a difference in degrees of mechanistic complexity). By contrast, enactive embodied cognition rejects Cartesianism by starting with the observation that there is in fact a categorical difference between inanimate physical entities and living bodily beings. While inorganic bodies passively and indifferently persist in space, living bodily beings are *autopoietic*. This means that they are in the business of actively and continually constituting their precarious autonomy as a bounded unified self via ongoing dynamic adaptive exchanges with their environment (Thompson 2007). From this enactive 4E perspective, the radical independency of the Cartesian mind is a phantasy: living beings maintain a precarious embodied autonomy through an ineluctable world-dependence. To capture the active meaningful connection to the world that living precarious embodied minds maintain, enactivists have introduced the term *sense-making*, which Hanne de Jaegher characterizes as follows:

“Exchanges with the world are inherently significant for the cognizer and this is a definitional property of a cognizing system; the creation and appreciation of meaning or sense-making in short. ... Sense-making is an inherently active concept. Organisms … actively participate in the generation of meaning in what matters to them; they enact a world” (Jaegher and Paolo 2007, 488).

Some 4E approaches have been criticized for implicitly assuming or even prioritizing a generic able-bodied cognitive agent in their accounts of cognition (for examples of such critiques see Protevi 2009; Van Grunsven 2020).^10^ By contrast, I see it as a central commitment of *enactive* 4E Cognition that it adopts a stance towards a person’s embodied life, disabled or otherwise, as anchoring them to a meaningful world through their particular history of ongoing self-constitution, which is reflected in their ways of moving and coping with their environment and their styles of embodied expressivity. Enactivism is committed to foregrounding that, even when someone’s world is a harder one to navigate, it is still *their* world with perceptual saliences that might matter deeply to them. De Jaegher explicates how this starting point opens up a different way of seeing and imagining the embodied lives of those who deviate from what is taken to be normal. Focused specifically on autistic embodiment, she writes:

“If autistic embodiment is intrinsically linked with autistic sense-making, we can hypothesize that many autistic people will find joy or significance in behaviors and embodied styles of sense-making that are considered ‘autistic…’ (9) … Rich patterns exist everywhere in the world, and many autistic people value them, care about them, even enjoy them. … People with autism … may feel that they will lose something salient if they (are made to) try to [ignore these patterns” (De Jaegher 2013, See also Manning 2016; Van Grunsven and Roeser 2022).

Note the stark contrast between this outlook and the technoableist proposal that the eradication of “autistic traits” with CRISPR_Cas9 counts as an appropriate “therapy” for (*read*: the eradication of) autistic people.

An enactive account of embodiment can begin to do justice to the intrinsic meaningfulness of different embodied expressions and worldly engagements. Still, that doesn’t yet explain the ease with which many of us are prone to bypass disabled forms of embodied life; seeing disabled living as somehow lacking in agency and meaningful sense-making. What I will now suggest is that the very same 4E account of the body that encourages us to reimagine it as the locus of lived experience and precarious sense-making, is also the account of the body that allows us to appreciate the depth at which ableism permeates and limits our imaginative outlook onto the world and the embodied lives seen as mattering. To expand upon this, I will turn to the notion of affordances, which is widely embraced in (enactive) 4E accounts of cognition and was coined by ecological psychologist J.J. Gibson (1979). Affordances are the perceivable possibilities for action that are available within an environment (or ‘ecological niche) to embodied precariously self-maintaining living animals. Put more concretely, a living animal’s morphology, its bodily needs, and its sensorimotor skills shape what it perceives as meaningful and relevant in its environment, while, at the same time, the environment co-shapes the needs and skills it develops. In the human case, what we perceive as meaningful and relevant is shaped through processes of enculturation, with dyadic and artefact-oriented embodied interpersonal engagements initiating us into a mind-bogglingly “rich landscape of affordances” (Rietveld and Kiverstein 2014). Gibson refers to this process of enculturation as an “education of attention,” which captures the normative process by which we learn which affordances are salient in our communities and how these affordances are to be appropriately responded to: “feel, this is how we stroke the cat,” “look, this is how we use a spoon or handle a book.”

When a person’s morphology, skills, and needs, jive with the dominant affordances in an ecological niche, then a person gains access to a world in which they are often able to effortlessly respond to relevant affordances. For instance, this morning, when I rode my cargo bike, racing to drop my kids off at school on time, it was in a pre-reflective effortless manner that my perception of the traffic light turning green afforded me with the cue to set my body in motion, push the pedal of the bike and quickly and appropriately cross the street. As I have argued elsewhere, this pre-reflective ‘world-familiarity,’ this sense of effortlessly knowing one’s way about in the world, is vital to one’s ability to flourish, preventing what some psychologists call ego-depletion (Van Grunsven 2021). What I want to emphasize here, is that the effortless world-familiarity enabled by our attunement to relevant affordances points to a duality at the heart of our experiential lives. On the one hand the notion of affordances captures our situatedness in a hospitable world of meaning, a world that we perceive as inviting a rich set of possibilities for action. On the other hand, our habitual responses to affordances can also close us off from other possible ways of seeing and imagining the world. In perceiving the traffic light as affording safe street-crossing I was *precisely not* perceiving it as affording a treacherous life-threatening action. However, when traffic lights aren’t equipped with audio signals, that *is* precisely what they afford to those of us who are blind.^11^ You might say that it is part of the logic of world-familiarity that one enjoys it robustly to the extent that one occupies the privileged position of being able to take it for granted.

From an enactive affordance-based perspective, then, not everybody enjoys equal access to the world understood as a space of familiar affordances. This confronts some of us with what Arseli Dokumaci calls a disabling *shrinkage* of affordances, where “what the environment affords diminishes in comparison to [x]. The environment becomes a less habitable place then [x], whatever [x] may be.” (2023, 53). From this perspective, assessing the optimal functioning of a living embodied human mind being requires that we move beyond a methodological individualism. Where our Cartesian sociotechnical imagination is prone to attribute the cause and badness of disability to alleged deficiencies located at the level of individual bodies and minds, an enactive affordance-based perspective asks us to adopt a broader relational stance. To the degree that we want to understand the functioning of embodied minds as confronting challenges that warrant technological intervention, we must look at the embodied-mind as situated in a particular environment with which it is ineluctably entangled (having to make sense of and with it), and that offers and can fail to offer a particular range of affordances.

Effortless rich world-familiarity–and shrinkage as its negative counterpart–operate at two intertwined levels. As I just illustrated with the traffic light example, it operates 1) at the level of the perceived possibilities for action afforded to a person by their environment, but 2) it also operates at the level of one’s visibility in social space; whether we are seen by others as skilled embodied agents who afford meaningful possibilities for interaction. After all, the education of attention that shapes how we see and respond to environmental affordances has a strong normative component, where what we come to see is not just how things *can* be coped with but how they are “canonically” *meant* to be coped with (Costall 1997; see also Dokumaci 2023). With that, the normativity of affordances affects how we evaluate people in their ongoing coping with the environment. Who we recognize as a skilled intentional interaction-affording person is co-shaped by acquired perceptual habits about what affordances we think matter and how we expect other body-minds to engage with those affordances.

**Fig. 1:**
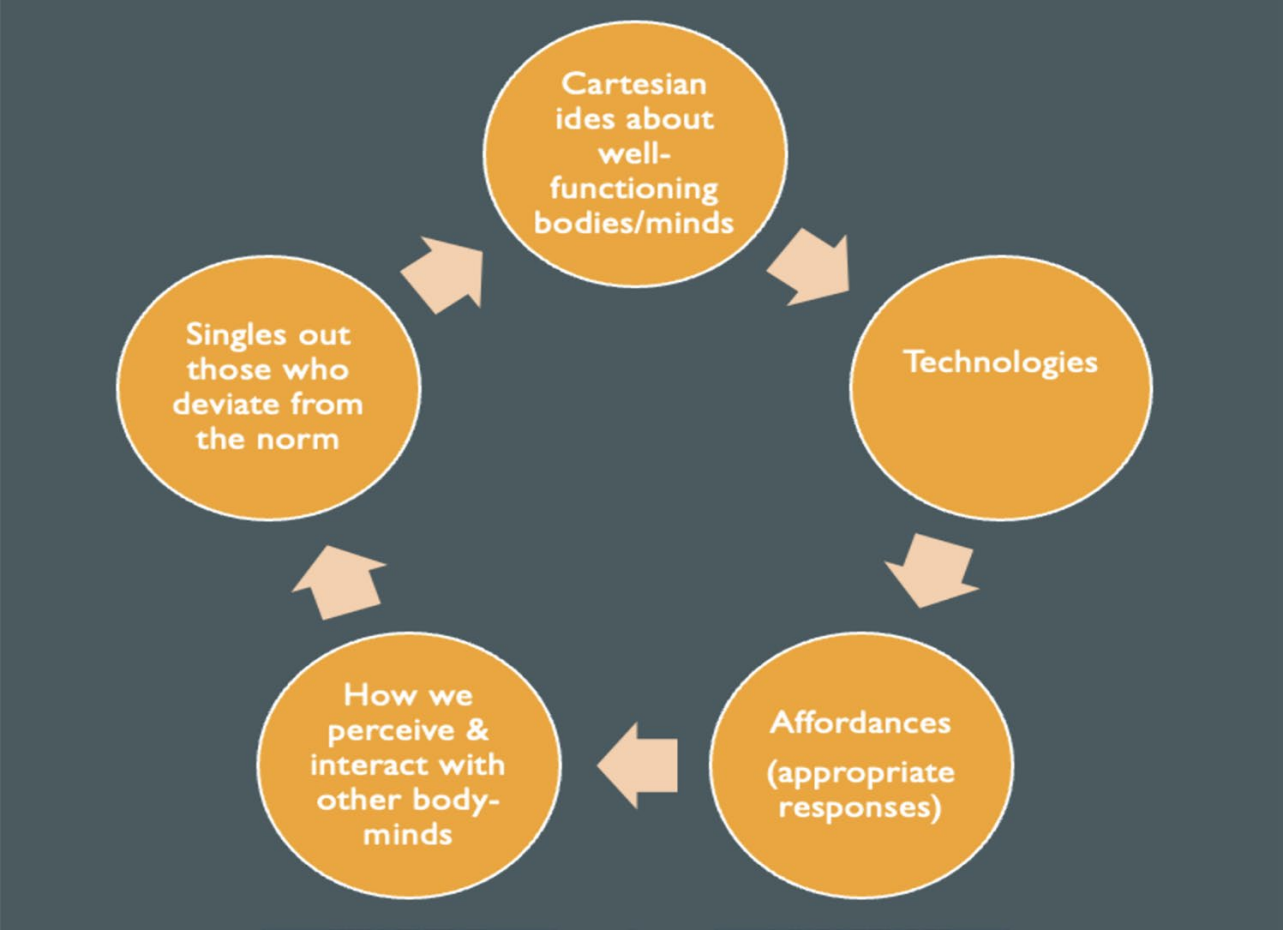
The vicious cycle of Cartesian Technoableism

You might say, then, that technoableism continually loops from the sociotechnical world in which we are embedded through our embodied minds and back again, creating a vicious cycle that those of us who enjoy effortless world familiarity may be hardly aware of. The cycle runs as follows: flawed Cartesian ideas about what well-functioning bodies and minds need and look like, as well as ideas regarding the promise of science and technology to intervene and solve ‘problems’ of bodily and minded malfunctioning, animate the endeavors of technologists and are engineered into the artefacts, technologies, and infrastructures that are designed and implemented. These artefacts, technologies, and infrastructures, once built and marketed, introduce canonical affordances into our lives, normatively shaping how we attend to and act within the world as well as how we evaluate others. When some of those others don’t comply with our habituated perceptual and agential expectations of the kinds of skilled actions afforded by the environment, going against the grain of the normativity of what we take the world to afford, we may quickly think something is wrong. Backed by Cartesian assumptions animating our Western sociotechnical imagination, our default stance is then to think that we need to bring someone into ‘the normal’ by intervening there where, from our standpoint, things look to be going wrong: at the level of ‘deficient’ individual body-minds (see Fig. 1).

An enactive affordance-based account of cognition can capture the workings of this vicious cycle, exposing its flaws, while also identifying areas for improvement. Towards this improvement, the enactive starting-point I am proposing suggests that our sociotechnical imagination must become grounded in a conception of the human body as, in the first instance, the site of precarious sense-making. It must be centered around the idea that such sense-making can take on a plurality of shapes due to the diverse nature of human embodiment, diverse histories of ongoing self-constitution, and diverse organism-environment interactions. It must be centered around the awareness that the dominant affordances recognized as salient within a given human society are stacked in favor of some and against others and that dual the logic of affordances can make it very difficult for those who enjoy effortless world-familiarity to see how this works in particular instances (recall my traffic light example, where my *seeing* the light as affording smooth and safe street-crossing also entailed my *not seeing* it as affording a treacherous activity).^12^ Because of this dual logic of seeing and not-seeing, an enactive reorientation of our sociotechnical imagination also points to the need to actively seek out different embodied sense-making perspectives, without which working to a more inclusive hospitable space of affordances would be practically impossible.

## Embracing Embodied Diversity in Our Sociotechnical Imagination for the Sake of all Human and Planetary Flourishing

In its most extreme form, and as reflected in at least one of the headlines we saw earlier, our Cartesian sociotechnical imagination aspires to a transhumanist eradication of diverse embodied perspectives. Hugh Herr who is a transhumanist amputee and professor at MIT co-responsible for the leadership of MIT’s 24 million dollar bionics center, emphatically embraces this vision, stating that:

“The world profoundly needs relief from the disabilities imposed by today’s nonexistent or broken technologies. We must continually strive towards a technological future in which disability is no longer a common life experience” (MIT News).^13^

In the final part of my paper, I will suggest that this stance is not only harmful to disabled people, it is quite conceivably also harmful for our efforts to find new realistic ways of supporting human and planetary flourishing as such. Unlike what transhumanists proponents of human enhancement may have us believe, our future in our volatile increasingly inhospitable world is not one of overcoming bodily limitations and disabilities. Rather, as Ashley Shew argues, it is disabled “along every axis of consideration … imagin[able]” (2023, 114). Not only is the future disabled “for each individual,” in the sense that “anyone who lives long enough can expect disability eventually;” it is also “disabled for humanity writ large” (climate change, pollution, and globalization-enabled pandemics are wreaking havoc on our precarious human bodies and the environments we inhabit); furthermore “the future is disabled for the planet itself,” (our Western Cartesian pursuit to dominate nature and plunder natural resources are shrinking innumerable ecological niches of other organisms); and, finally, Shew argues, the future is even “disabled cosmically,” where a bet on extraterrestrial survival would require reckoning with the fact that “space as an environment is not suited to any human bodies.” (2023, 118).

While Shew argues, convincingly, that the future is almost certainly disabled, she adds that there isn’t much else about the future that we can state with certainty. In fact, in a world rapidly changing, dealing with *un*certainty becomes increasingly important (see also Rietveld 2022). As it so happens, Shew points out, disabled people are “experts at uncertainty,” coping with a world that is in many ways unprepared, unaccommodating, or even hostile towards disabled embodiment:

“When we ask for the ability to live with uncertainty, we are asking to learn “the fine art” of being disabled. To grapple with the uncertain future, we would be well served to listen to disabled experts. The experiences and authentic stories of disabled people give us a lot of insight into our technological futures. Disabled people have expertise in navigating worlds not built for us–worlds that are often actively hostile to us” (2023, 123).

“The fine art of’ of being disabled.” Dokumaci strikes a similar tone in her book *Activist Affordances*, in which she argues, among other things, that disabled people who inhabit a world characterized by a shrinkage of possibilities for action are forced into a position of *creative performance-like sense-making*, of imaginatively enacting “activist affordances,” which Dokumaci defines as “possibilities for action that are almost too remote and therefore unlikely to be perceived, and yet are perceived and actualized through great ingenuity and effort to ensure survival” (6). Activist affordances are, in her words,

“born of a need and a desire to go beyond the limits of a narrowed environment … in which our sick, impaired, injured, painful, hurting, dying, and nonstandard bodies/minds are not recognized or welcomed as they are … [Activist affordances] … imagine a then and there … in which the world becomes inhabitable otherwise. […] The activist affordances that disabled people have long mastered can perhaps be a way of addressing the pressing question of how to negotiate a shrinking planet with diminishing recourses (2023, 246-7 & 252).

If Dokumaci and Shew are right, we all stand to benefit from attending to the kinds of embodied know-how and creative world-making that disabled people and communities have to offer, but that our Cartesian sociotechnical imagination is prone to overlook or dismiss. After all, if disability is marked by a shrinking relationship to one’s environment, and if the destruction of the planet–the environment shared by all living beings–will inevitable confront all of us with such shrinkage, then the experience of disability, as shrinkage, is something all of us will be grappling with (though, undoubtedly, the degree to which we will confront this will vary in accordance with various forms of privilege). If this is the case, then those of us we recognize as disabled, and who have extensive experience creatively navigating shrinking, hostile, and uncertain environments, are vital source of authority, both when it comes to coping with conditions of uncertainty and environmentally imposed limitations and when it comes to imagining different ways to live. The Cartesian technoableist view of disabled people as deficient passive non-agents, who lack an epistemically authoritative perspective on how to navigate shrinking environments, and whose body-minds need fixing by experts in applied science, is not only ontologically flawed and ethically harmful, it is also empirically false. There is a rich history of disabled people creatively tinkering with hostile socio-technical environments and imaginatively adjusting their worlds and body-minds so as to imagine and enact more accessible worlds. This is often done without access to bountiful resources and fancy technologies, relying more so on maintenance and community instead (Hamraie and Fritsch 2019; Shew 2023). Problematically, though, if we think of disability as a problem to be solved with technoableist methods and techniques, we close ourselves off from the voices of disabled people and from seeing disability as a method for addressing a larger crisis that is inevitably underway.

In a world undergoing devastating life-threatening changes, caused by a centuries-long Western Cartesian desire for mastery over nature, we need to tap into much needed alternative modes of embodied perceiving, acting, and living. What I have been suggesting, is that this requires a reimagining of the human body in a way that embraces embodied diversity and that encourages us to learn from the embodied imaginations of disabled people—people who are experts at coping with uncertainty and at creatively surviving in a world characterized by shrinking possibilities for action; a world that is becoming increasingly hostile to all forms and shapes of life. Alternative, creative visions for how to live and value precarious embodied environment-dependent human life are available all around us—not just through the voices and expertise of disabled people and critical disability studies scholars, but also via feminist, queer, and decolonial perspectives.^14^ Indeed, as Joan Rothschild already warned in 1980 in “a Feminist Perspective on Technology and the Future:”

We are locked into an anti-ecological, anti-humanistic, exploitative technology. … A feminist perspective can guide us to a technology that will create, not destroy. But we must avoid the trap of … scientists and technologists whose denial of the human, pursuit of false objectivity, and drive to conquer nature lead to an irrational impasses. What is the level of rationality of a society that depletes finite resources, that poisons the atmosphere, that develops a weapon that kills living things but spares inanimate objects?” (66 & 72)

Despite Rothschild’s hope for a feminist perspective to “guide us to a technology that will create, not destroy,” a quasi-Cartesian sociotechnical imaginary continues to prevail in Western science and engineering contexts. This imaginary is reflected in, as well as fed by, hyped interventionist narratives about emerging technologies ‘fixing’ malfunctioning disabled body-minds, where it is both through what interventionist-minded technologists *see* and *don’t see*, *do* and *don’t do* (targeting individual body-minds while ignoring the wider shared environment), that they stack our sociotechnical niches in favor of some at the expense of others. As I have argued, the affordances emergent within these constructed sociotechnical niches loop back into habituated normative patterns of seeing and acting, which, in turn, feed into the vicious cycle of Cartesian technoableism. Given the pervasive way in which technoableism continues to take root, we need all the resources we can get to arrive at “ideas and models for an alternative technological future” (Rothschild 1981, 68). We need the expertise and know-how of those whose embodied lived experiences rub against the habituated ways in which we have come to see, organize, and (de)value certain people, forms of life, and the environment more generally. But we also benefit from conceptual-theoretical resources that can replace the quasi-Cartesian image of body and mind animating our sociotechnical imagination with an alternative ontology that can inform and guide the developments of new technologies, enabling engineers to help open up new affordances and ways of living.^15^ I have presented an enactive account of living embodied beings as sense-makers as a fruitful candidate for this job. While much work remains to be done to fill in the details, I hope my proposal contributes to the growing effort, within 4E Cognition, to better understand how the tools and infrastructures that we engineer can be hostile to human and planetary flourishing. Indeed, I hope my proposal can contribute not only to how we *understand* this, but also to how we (particularly today’s and tomorrow’s engineers) might *respond* to this. I am thus, perhaps, retaining a bit of Cartesian technological optimism, holding that the theoretical ontological constructs we work with can have far-reaching real-life consequences and that this can be for the worse, but, perhaps, also for the better.

## Footnotes

1 In fact, as I argue in Van Grunsven 2015 (chapter 3), Descartes’ account of the mind–body unit, as developed in the 6th meditation, gestures at an affordance approach to perception that points back to the body as the site of lived experience.

2 Indeed, even Descartes’ contemporaries largely rejected the specifics of his account including, perhaps, the Descartes of the 6th Meditation himself (see Van Grunsven 2015, Chapter 3).

3 https://www.theguardian.com/lifeandstyle/2012/aug/12/exoskeletons-help-disabled-people accessed on September 29th 2023.

4 https://www.newsweek.com/scientists-edit-autism-genes-traits-texas-crispr-research-mice-997948 accessed September 29th 2023.

5 https://venturebeat.com/ai/its-time-to-embrace-the-tech-that-could-confront-the-crisis-in-aging/ accessed July 3.^rd^ 2024.

6 Erin Manning worries about this trend when she writes: “anything that makes us less independent in the eyes of a world that takes intentionality and volition as a normative standard tends to decrease our perceived value as contributors to society” (2016, 21).

7 https://www.businessinsider.com/robotic-suit-world-cup-2014-6?international=true&r=US&IR=T Accessed 29th September 2023.

8 https://www.youtube.com/watch?v=PCWIGN3181U Accessed 29th of September 2023.

9 This mismatch operates at different levels, creating misconceptions about the degree to which disability is bad, the reasons for it being bad, and the actual needs, desires, and abilities that disabled people have.

10 As an anonymous reviewer helpfully pointed out to me “It seems plausible … that there is an implicit technoableism at work in design that has been important for the development of extended cognition, especially in works by Donald Norman. *The Design of Everyday Things* (2013) presupposes a generic (and implicitly but clearly not disabled) subject as the measure of good design, and that ideal has informed much thinking about [cognitive and affective] scaffolding.” Interestingly, later in life, Norman became emphatically critical of the rampant ageism imbued in much of the design of everyday things (2019). Of course, at that point his influence on our thinking about scaffolding from the perspective of a generic non-disabled body had already become entrenched.

11 I borrow this example from Dokumaci (2023).

12 Another way to capture this, as Mireille Hildebrandt argues, is that this stems from the fact that the normative force of technological artefacts, in contrast with, for instance, the normativity of legal statues and laws, “does not depend on deliberate inscription” but on our embodied habitual ways of knowing our way around our sociotechnical niches, which “makes it hard to discern and contest the normative constraints insofar as we are not used to detecting unintended normative implications in the technologies of our own making” (2017, 605–6).

13 https://news.mit.edu/2021/new-bionics-center-established-mit-24-million-gift-0923 (accessed May 31st 2024).

14 This is not a coincidence. As some argue, ableism is inextricably entangled with “anti-Blackness, eugenics, misogony, imperialism, and capitalism … You do not have to be disabled to experience ableism.” (Talila ‘TL’ Lewis on TL’s website: https://www.talilalewis.com/blog/january-2021-working-definition-of-ableism). Critiques of the attitude of domination built into the Cartesian (and the broader rationalistic Enlightenment) project are also abound in critical theory (e.g. in Horkheimer & Adorno 2002) and in continental philosophy of technology (e.g. Jonas 1979; Ellul 2021).

15 Some examples of how enactive insights on human embodiment and (participatory) sense-making can provide normative design requirements for technology are already out there (e.g. Fletcher-Watson et al. 2018; Van Grunsven & Roeser 2022).
